# Forgotten Intrauterine Device Causing Postmenopausal Endometritis: A Case Report

**DOI:** 10.7759/cureus.97856

**Published:** 2025-11-26

**Authors:** Irem Dogan, Ahmet C Demirsah

**Affiliations:** 1 Department of Obstetrics and Gynecology, Bolvadin State Hospital, Afyonkarahisar, TUR; 2 Department of Radiology, Bolvadin State Hospital, Afyonkarahisar, TUR

**Keywords:** endometritis, evaluation of postmenopausal bleeding, intrauterine device, postmenopause, uterine hemorrhage

## Abstract

Intrauterine devices (IUDs) are among the most widely used long-acting reversible contraceptives. Although generally safe, forgotten or retained IUDs, beyond their intended duration, can lead to complications, particularly in postmenopausal women.

We describe an 85-year-old gravida 4, para 4 woman, postmenopausal for 40 years, who presented with yellowish vaginal discharge, groin pain, and vaginal bleeding. Transvaginal ultrasound demonstrated irregular echogenicities within the endometrial cavity, while transabdominal ultrasound showed an echogenic structure with posterior acoustic shadowing, suggestive of a retained IUD. A computed tomography (CT) scan performed six months earlier had also revealed the presence of the device, but no intervention was done at that time. The IUD was successfully removed with forceps, despite the absence of retrieval strings, with purulent discharge noted. Histopathology of endometrial curettage confirmed acute and chronic endometritis with abscess formation, but no evidence of malignancy. Symptoms resolved following removal and antibiotic therapy. Retained IUDs in elderly postmenopausal women may remain undetected for decades and present with bleeding, infection, or incidental imaging findings. Prolonged retention is associated with infectious complications and technical difficulties during retrieval. Guidelines recommend removal once contraceptive need has ceased. This case emphasizes the importance of identifying and removing forgotten IUDs in postmenopausal women to prevent infection, ensure accurate imaging interpretation, and allow safe retrieval.

## Introduction

Intrauterine devices (IUDs) are among the most widely used contraceptive methods worldwide, due to their high efficacy, cost-effectiveness, and reversibility. Since the introduction of the Lippes Loop in the early 1960s, IUDs have been utilized extensively as long-acting reversible contraceptives [1,2]. While generally safe, their prolonged use or retention beyond the intended duration may lead to complications. Historically, early inert IUDs, such as the Lippes Loop, were designed for long-term implantation and were therefore often left in place until menopause. Consequently, many women retained these devices for decades, with some presenting even in the postmenopausal period with a Lippes Loop still in situ - either intentionally or because it had been forgotten. However, forgotten or neglected IUDs have been reported even decades after insertion, with some cases remaining asymptomatic, while others present with pelvic pain, abnormal bleeding, infection, or more severe sequelae, including uterine perforation and device migration [1,3,4].

In this report, we present a case of a long-retained IUD discovered decades after insertion and review the literature on prolonged IUD retention and its potential complications.

## Case presentation

An 85-year-old gravida 4, para 4 woman, postmenopausal for 40 years, presented to the gynecology outpatient clinic with a three- to four-day history of yellowish vaginal discharge and groin pain, along with two weeks of intermittent vaginal bleeding that stained her underwear. She denied any passage of clots. The patient’s medical history included coronary artery disease, type 2 diabetes mellitus, and hypertension, for which she was receiving oral antidiabetic agents, antihypertensive drugs, and anticoagulant therapy. She reported no family history of gynecologic malignancy, and no prior use of hormone replacement therapy. Additionally, she had no history of sexually transmitted infections, pelvic trauma, or previous surgical procedures. Gynecologic history was notable for menarche at age 12 and menopause at age 47. The patient was illiterate and resided in a rural area of Central Western Anatolia. She reported that she had not attended routine gynecological check-ups.

The patient reported that an IUD had been inserted following her fourth delivery, 50 years earlier. She could not recall any subsequent removal procedure and stated that she was unaware that the IUD should be retrieved after menopause.

**Figure 1:**
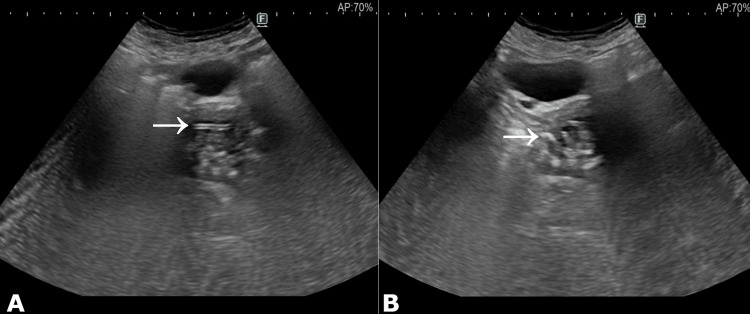
Transabdominal ultrasound images of the pelvis (A) Sagittal plane and (B) axial plane demonstrating an echogenic linear structure (arrows) with posterior acoustic shadowing within the endometrial cavity, consistent with an intrauterine device.

She was afebrile and denied pelvic pain or systemic symptoms. The patient’s BMI was 25.8 kg/m². On physical examination, the external genitalia appeared normal. Speculum examination showed an atrophic cervix, without active vaginal bleeding or leucorrhea. Cervical cytology (Pap smear) was performed for screening purposes. Bimanual examination revealed a normal-sized, tender cervix with no adnexal masses. Transvaginal ultrasonography demonstrated an endometrial thickness of 4 mm and an echogenic serpentine structure with posterior acoustic shadowing in the endometrial cavity, consistent with a retained IUD. Both ovaries were atrophic, and no adnexal pathology was observed. Transabdominal ultrasonography similarly showed an echogenic structure with posterior acoustic shadowing in the endometrial cavity, supporting the diagnosis of a retained IUD (Figure 1).

**Figure 2:**
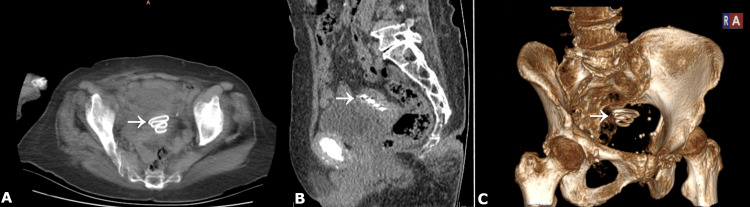
Computed tomography (CT) images of the pelvis demonstrating the intrauterine device (A) Axial minimum-intensity projection, (B) sagittal plane reconstruction, and (C) three-dimensional volume-rendered image confirm the intrauterine location of the device (arrows) within the endometrial cavity, without evidence of extrauterine migration, perforation, or involvement of adjacent pelvic organs.

The patient reported no symptoms suggestive of IUD-related complications, apart from postmenopausal bleeding and occasional groin pain. Review of prior imaging showed that an abdominopelvic computed tomography (CT) scan (Figure 2), obtained six months earlier during an admission for evaluation of chronic constipation, had also demonstrated the IUD. 

**Figure 3:**
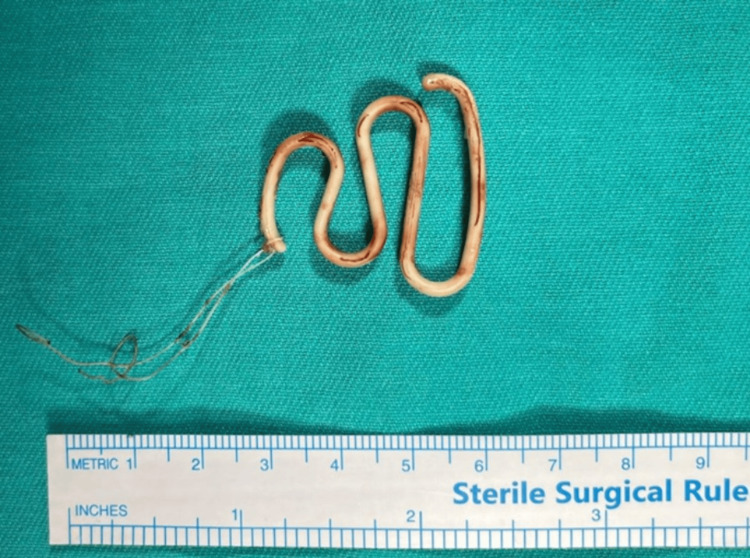
Removed intrauterine device Removed intrauterine device (Lippes Loop), retrieved intact with attached strings following removal from the uterine cavity. A sterile surgical ruler is shown for scale.

Although no retrieval strings were visible, the device was successfully removed intact in one piece using alligator forceps (Figure 3).

A purulent discharge was noted during extraction, prompting empiric antibiotic therapy. Endometrial curettage was performed using a Pipelle device.

**Figure 4:**
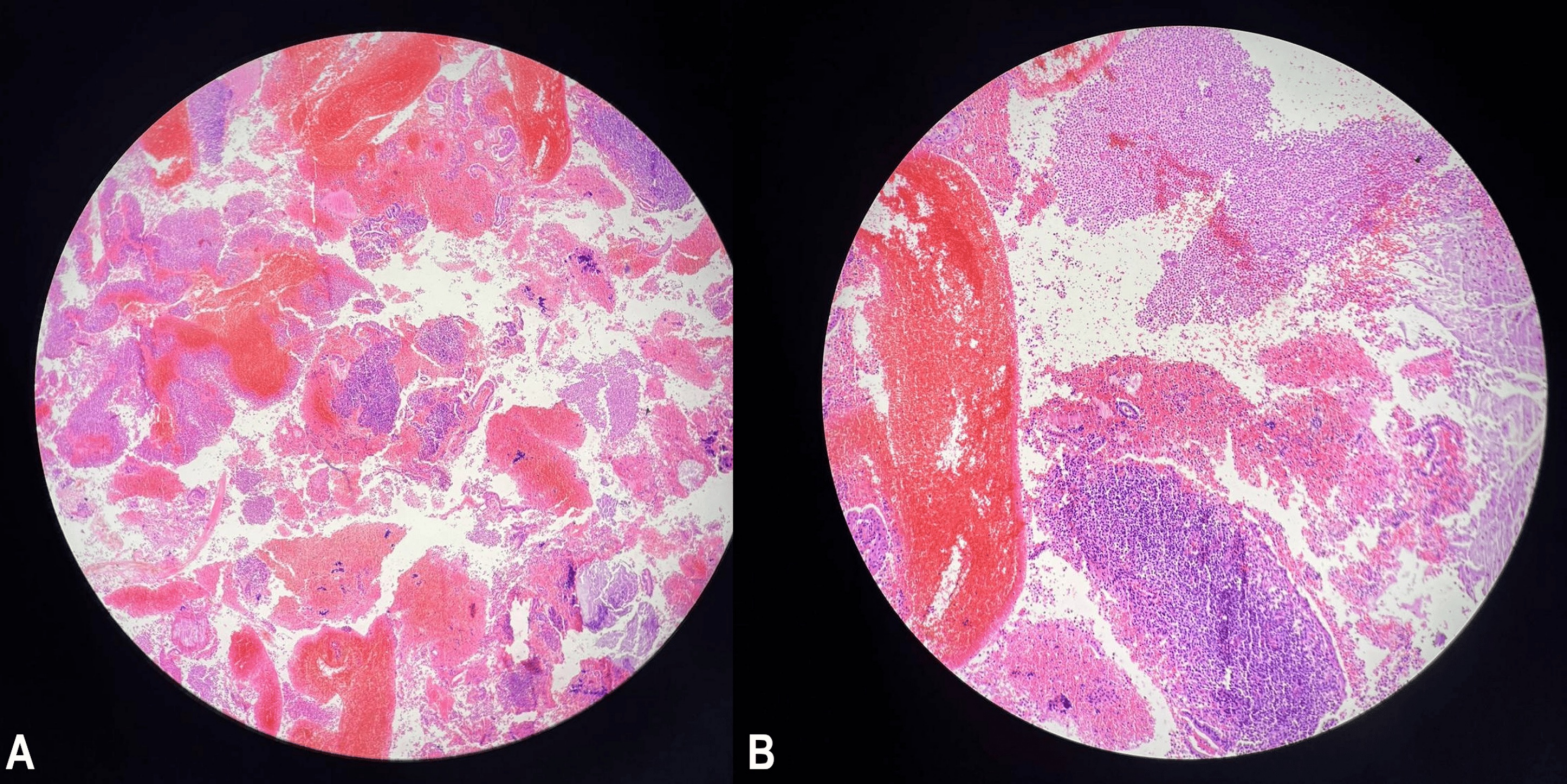
Histopathological examination of the endometrial tissue (A, B) Microscopic images (hematoxylin and eosin stain) demonstrate acute and chronic inflammatory cell infiltration with hemorrhagic fibrinous material, consistent with acute and chronic endometritis. No evidence of malignancy is observed.

Histopathological analysis of the endometrial sample revealed acute and chronic endometritis, with abscess formation and extensive hemorrhagic fibrinous tissue. No evidence of malignancy was identified (Figure 4).

**Figure 5:**
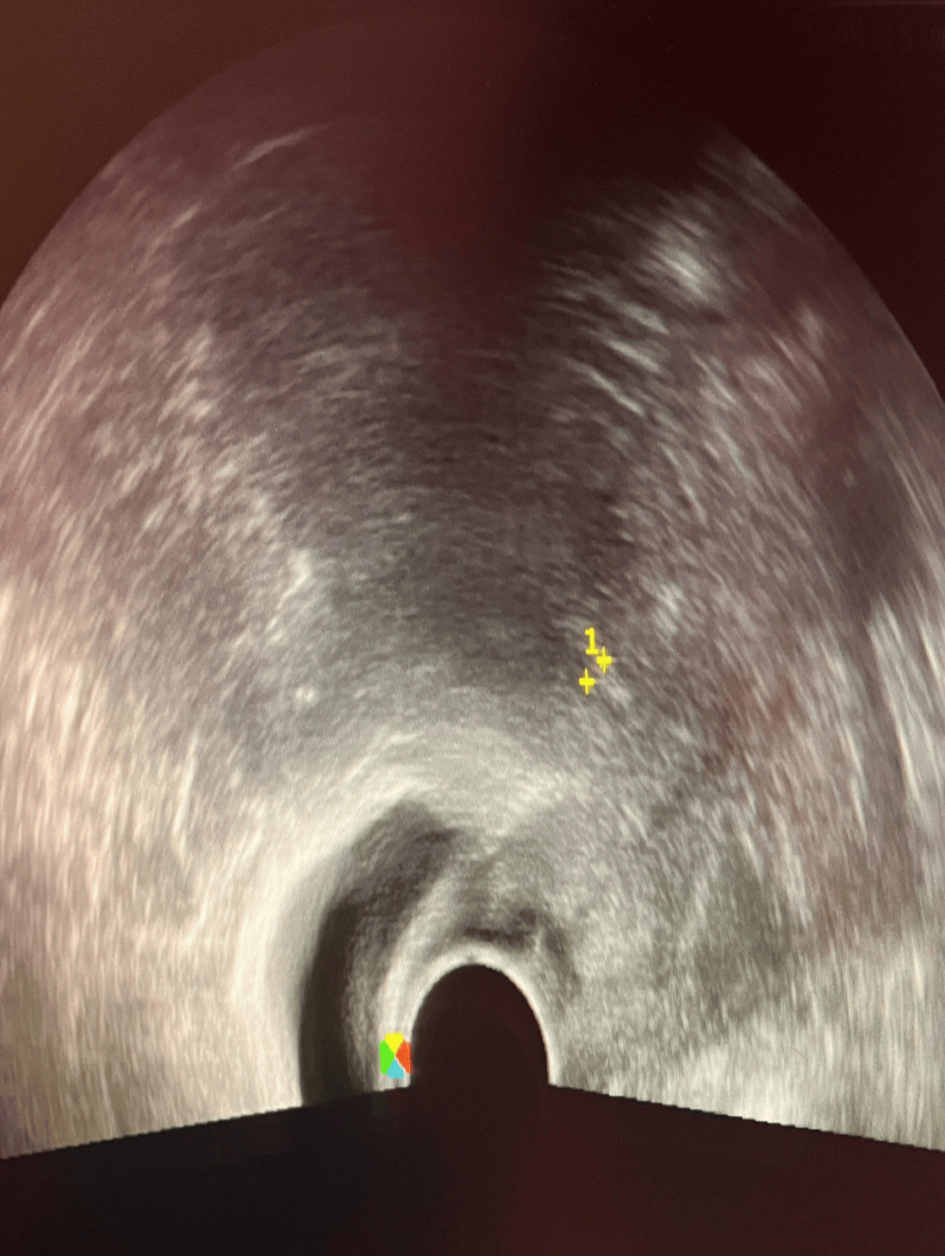
Follow-up transvaginal ultrasound image Post-removal sonogram demonstrates a regular, thin endometrial lining with homogeneous echotexture, measuring approximately 3 mm in thickness. No residual intrauterine device fragments or endometrial abnormalities are observed.

Cervical cytology revealed no evidence of intraepithelial lesion or malignancy. Post-extraction of the IUD, the patient remained clinically stable, without recurrence of symptoms, and the vaginal bleeding resolved. Subsequent transvaginal sonography demonstrated a normal endometrial lining, with adnexa of normal appearance (Figure 5).

## Discussion

Long-retained IUDs have been associated with various clinical presentations, particularly in postmenopausal women. Numerous case reports have highlighted that symptoms such as pelvic pain, abnormal uterine bleeding, and genital tract infections may manifest decades after insertion if the device is not removed at the appropriate time [1,2]. The Faculty of Sexual & Reproductive Healthcare (FSRH) guideline on intrauterine contraception specifically recommends that intrauterine contraceptives should normally be removed once they are no longer required, rather than being left in situ indefinitely [5].

Among the complications associated with long-retained IUDs, uterine perforation is the most frequently reported, although adjacent structures such as the rectum and urinary bladder may also be affected. According to the WHO IUD report, a displaced IUD should be removed due to the potential risk of complications such as bowel injury, persistent pelvic pain, and infertility [6]. However, there are exceptional reports in the literature describing conservative follow-up of peritoneally located IUDs, particularly in asymptomatic patients [7].

Another important consideration in the retention of an IUD is the risk of pelvic inflammatory disease. Although infection risk is lower in this age group due to hormonal and anatomical changes, a chronically retained IUD may still act as a nidus for infection, as demonstrated in our case with histopathological evidence of acute and chronic endometritis [1,3].

Furthermore, in elderly women, the presence of an IUD may complicate gynecological imaging by generating artifacts or signal voids that can obscure adjacent pelvic structures. Previously, a study demonstrated that both copper-containing and non-metallic IUDs are considered safe during magnetic resonance imaging (MRI); however, it also noted that metallic components, particularly copper, can generate magnetic susceptibility artifacts that may reduce diagnostic accuracy for the uterus and surrounding pelvic structures. This limitation is especially relevant in elderly patients, in whom alternative gynecological pathologies, such as endometrial or myometrial lesions, are more likely to be investigated with imaging modalities [8].

Additionally, recent studies have noted that prolonged retention may lead to technical difficulties during removal [9]. In our patient, despite the absence of visible strings, the IUD was successfully retrieved in one piece with forceps.

Limited medical literacy, particularly in rural or underserved populations, may contribute to unintentional long-term IUD retention. Patients may be unaware of the device’s lifespan or the importance of removal after menopause. Clear patient education at insertion and during routine visits is essential, and physicians play a key role in ensuring that patients understand proper IUD management to prevent avoidable complications.

Overall, this case emphasizes the importance of identifying and removing retained IUDs in postmenopausal women, not only to alleviate symptoms but also to prevent infectious and mechanical complications, facilitate accurate imaging, and avoid technical challenges during removal.

## Conclusions

Retained IUDs can remain asymptomatic for decades but carry risks of infection, bleeding, and diagnostic challenges in postmenopausal women. Timely recognition and removal are essential to prevent complications, as demonstrated by the successful retrieval of the device and resolution of endometritis in this case.
